# 阵发性睡眠性血红蛋白尿症克隆筛查及补体抑制剂治疗监测中国专家共识（2024年版）

**DOI:** 10.3760/cma.j.cn121090-20230927-00145

**Published:** 2024-02

**Authors:** 中华医学会血液学分会红细胞疾病（贫血）学组

**Keywords:** 阵发性睡眠性血红蛋白尿症, 筛查, 诊断, 补体抑制剂, 治疗监测, Paroxysmal nocturnal hemoglobinuria, Screening, Diagnosis, Terminal complement inhibitor, Treatment monitoring

## Abstract

阵发性睡眠性血红蛋白尿症（paroxysmal nocturnal hemoglobinuria，PNH）是一种罕见的造血干细胞异常克隆性疾病，以血管内溶血性贫血、血栓形成和外周血细胞减少为主要表现，呈慢性进展性病程，严重者可危及生命。补体抑制剂是治疗PNH溶血相关症状的一线推荐药物。随着补体抑制剂领域的快速发展，加强对PNH的筛查、快速诊断，判断需要用补体抑制剂治疗的患者，在补体抑制剂治疗过程中监测突破性溶血、血管外溶血等，对患者的生存、生活质量改善有着重要意义。为促进PNH临床诊疗的规范，本共识参考国内外最新指南和文献，荟萃国内外最新研究成果，并结合专家团队经验，聚焦 PNH筛查原则、PNH克隆检测意义、末端C5补体抑制剂治疗后监测等问题，旨在为PNH的筛查、诊断和补体抑制剂时代的治疗监测提供参考意见。

## Abstract (en)

Paroxysmal nocturnal hemoglobinuria (PNH) is a rare clonal disease with abnormal hematopoietic stem cells that causes intravascular hemolytic anemia, thrombosis, and peripheral blood cytopenia. It has a chronic progressive course and can be fatal in severe cases if not treated aggressively. Complement inhibitors are the first-line recommended treatment for hemolysis-related symptoms of PNH. With the rapid development of new complement inhibitors, it is critical to quickly screen and confirm the diagnosis, identify patients with complement inhibitor indications, and monitor breakthrough hemolysis and extravascular hemolysis during complement inhibitor therapy. Drawing on the most recent guidelines, works of literature, and meta-reviews from around the world, as well as combining with experience from the experts, this consensus focused on PNH screening principles, the significance of PNH cloning detection, and post-treatment monitoring of terminal complement inhibitors, which may contribute to a better understanding of diagnosis and treatment monitoring in the era of complement inhibitors.

阵发性睡眠性血红蛋白尿症（PNH）是一种获得性、发生于多能造血干细胞层面的糖化磷脂酰肌醇A类（PIGA）基因体细胞突变引起的罕见疾病。PIGA编码糖基磷脂酰肌醇（GPI）生物合成第一步所需的酶，PIGA突变导致患者造血干细胞及向下分化的血细胞表面缺乏GPI锚定蛋白，其中，两种必需的补体调节蛋白CD55和CD59的缺乏使PNH红细胞易发生补体介导的溶血[1]。PNH主要表现为反复发作的血管内溶血（IVH）、血栓形成倾向和不同程度的骨髓衰竭，国际PNH工作组（I-PIG）将PNH分为三类[1]–[2]：①经典型PNH：有典型的溶血和血栓形成表现；②合并其他骨髓衰竭性疾病（BMF）的PNH：如再生障碍性贫血（AA）或骨髓增生异常肿瘤（MDS）；③亚临床型PNH：患者有少量PNH克隆，但没有溶血和血栓的临床和实验室证据。

PNH虽然是一种良性疾病，但在传统的治疗手段下，患者10年总生存（OS）率仅70.7％～77.6％，生存率及生活质量亟待提高。PNH患者因慢性溶血可引起疲劳、血栓、肾功能不全、疼痛、意识障碍、呼吸困难等多种临床表现，首诊科室往往并非血液科，易被误诊、漏诊，导致随病程推移出现器官功能障碍[3]–[4]。此外，在BMF中，如何理解PNH克隆存在的意义、如何进行合理监测和随访也需规范。

靶向末端补体C5的抑制剂依库珠单抗，是首个被美国食品药物管理局（FDA）和欧洲药品管理局批准用于治疗溶血性 PNH的补体抑制剂，目前已成为溶血性PNH 的首选治疗药物[3]，2022年底在中国获批上市。尽管以依库珠单抗为代表的第一代补体抑制剂取得了很好的疗效，但如何及时判断补体抑制剂效果不佳的可能原因，监测突破性溶血（BTH）和血管外溶血（EVH）[5]–[7]等尚无相关的共识或指南。中华医学会血液学分会红细胞疾病（贫血）学组组织相关专家，结合PNH筛查、诊断、随访和补体抑制剂使用管理的最新文献，形成本共识，为临床提供参考。

一、PNH克隆筛查

（一）需要进行PNH筛查的人群

PNH误诊率极高，首次诊断即确诊的患者仅7％，起病一年内确诊者仅35.5％[4]。确诊AA、MDS和不明原因贫血的患者，如进行PNH筛查，均有一定比例确诊为PNH[8]–[11]。对于出现血小板减少、不明原因腹痛（平滑肌张力障碍）、IVH或血栓形成的患者也应怀疑PNH，应及早进行外周血PNH克隆流式检测筛查，以免延误治疗。此外，PNH患者出现血液科以外的其他相关症状，首诊于其他科室，可能造成诊断延迟[9]–[12]，消化内科、神经内科、皮肤科、心内科、呼吸科、急诊科、血管外科、基本外科等，都可能成为患者的首诊科室，应加强多学科协作，提升相关科室对该病的认识（表1）。

（二）在BMF中筛查和随访PNH克隆的意义

在大多数健康个体中可以检测到0.001％～0.005％的微小PNH克隆，约1％健康个体中可能出现0.02％～0.03％的稍大PNH克隆，然而，在没有免疫攻击的背景下，PNH细胞一般不会发生扩增。在免疫介导的骨髓衰竭，如AA、低增生MDS（h-MDS）患者中，PNH克隆常常存在。

具有全血细胞减少、骨髓增生低下等BMF表现特征的疾病可由不同的病因引起，AA的诊断需要排除其他类似表现的疾病，对儿童和年轻患者特别要排除遗传性BMF（IBMF）。由于PNH细胞的克隆扩增与免疫机制介导的骨髓衰竭关系密切，PNH克隆的检出可为AA患者的免疫介导机制提供重要的诊断线索[9]。

既往研究指出，在BMF中检出PNH克隆对诊断免疫介导的AA更有帮助，也有助于排除如范可尼贫血、先天性角化不良等IBMF，因为PNH克隆极少在IBMF中出现[2]。无论克隆大小，PNH粒细胞克隆的出现，对免疫介导的AA阳性预测值约为100％；而在130多例IBMF患者中，却没有发现PNH克隆或PIGA突变。免疫介导的AA与IBMF相比，发现PNH克隆的概率高25倍。因此，PNH克隆的出现，为诊断免疫介导AA提供了特异性的额外支持。值得注意的是，PNH阴性并不能排除免疫介导的AA诊断，不应通过未检出PNH克隆来排除AA。

**表1:** 应进行阵发性睡眠性血红蛋白尿症（ PNH）筛查的适宜人群

临床特征	适宜人群及建议
血细胞减少症	原因不明的血细胞减少症，尤其是全血细胞减少、考虑进行骨髓检查的患者
骨髓衰竭性疾病	怀疑AA/h-MDS或伴有溶血证据的患者，建议在诊断时进行PNH筛查，并至少每 6 个月筛查1次
血栓形成	不明原因血栓形成，伴有/不伴有未知原因的溶血患者；腹腔内/颅内/真皮层等特殊部位的动静脉血栓形成的患者；伴有全血细胞减少的易栓症患者；抗凝效果不佳的年轻患者
不明原因的溶血性贫血	Coombs阴性溶血性贫血的患者；血红蛋白尿/尿含铁血黄素阳性的患者；伴肾功能不全的溶血性贫血的患者
缺铁性贫血	补铁治疗效果不佳，LDH水平升高，间接胆红素升高或血红蛋白尿的患者

注 AA：再生障碍性贫血；h-MDS：低增生骨髓增生异常肿瘤

PNH克隆的出现，还与BMF的预后相关。在大多数关于AA预后的研究中，PNH克隆（无论克隆大小）与免疫抑制治疗反应的改善和总体预后的改善相关。存在PNH克隆患者免疫抑制反应疗效好的原因尚不完全清楚，但部分原因可能是PNH克隆的存在，使免疫介导的AA诊断更准确，从而可能对免疫抑制剂疗效更佳[13]。PNH克隆还与AA患者较低的MDS进展率相关。PNH克隆也可出现在h-MDS患者中，但较AA罕见，同样提示可能是免疫介导机制和对免疫抑制剂疗效较好。PNH克隆在其他恶性程度更高的髓系肿瘤（如骨髓增殖性肿瘤、急性髓系白血病等）中极为罕见。

（三）PNH克隆大小与溶血相关症状的关系

在PNH患者中，PNH克隆的大小与临床表现也有一定关联。通过流式细胞术检测，发现在PNH患者中，PNH克隆呈现“双峰”分布，即有较小PNH克隆和较高PNH克隆的患者较多，而存在中等大小PNH克隆的患者较少。较小PNH克隆，往往在亚临床型或以骨髓衰竭为主要表现的患者中出现。而那些以经典PNH表现为主者，PNH克隆往往较大，平均大小超过70％[9]。值得注意的是，由于不同的PNH亚型之间可以相互转换，造成临床特征不典型[14]–[16]。临床上，不能简单将PNH克隆大小直接对应临床表现。

PNH患者启动补体抑制剂治疗取决于多个因素，包括PNH克隆大小、溶血症状和疾病负担。以骨髓衰竭为主要临床表现的患者平均PNH克隆较小，较少出现PNH相关典型症状，很少能从补体抑制剂治疗中获益。因此，在有骨髓衰竭表现的PNH患者中，启动补体抑制剂治疗时，应仔细评估患者贫血的病因，包括了解网织红细胞、白细胞及血小板计数[2]。

经典型PNH往往有较大的PNH克隆（通常>50％），常呈现较高的疾病负担表现，如腹痛、疲劳、呼吸困难、食管痉挛、勃起功能障碍、神经功能障碍、肾功能不全、胆石症及各种血栓栓塞表现，包括不寻常部位血栓[3]–[4]。PNH患者血栓形成的危险因素包括粒细胞PNH克隆大小超过50％，LDH≥1.5倍正常上限（ULN），PNH症状负担重以及既往的血栓事件等。红细胞PNH克隆和粒细胞PNH克隆超过一定数值，可在一定程度上预测LDH升高；此外，粒细胞PNH克隆大小每增加10％，血栓形成约增加1.6％。尽管如此，PNH克隆大小、溶血和血栓风险之间的关系并不完全是线性的[16]。

（四）BMF中PNH克隆的监测[17]–[20]

PNH克隆也可能在AA或h-MDS病程的后期出现，定期通过流式细胞术监测PNH克隆，有助于早期发现PNH克隆演变；同时，在病程后期发现的PNH克隆，将支持在诊断时不易明确的BMF患者中的免疫介导因素。对BMF患者出现血栓形成或溶血等临床表现者，如果初始检测未显示 PNH 克隆，后续也应进行随访检测。诊断时已发现小克隆的AA或MDS患者，连续监测也很重要，因为患者可能从伴有小PNH克隆的AA或MDS，进展为溶血性 PNH，及时发现，可以迅速加用针对性治疗[14]。

即使是诊断为PNH的患者，也需要对PNH克隆进行定期监测。其中一些患者的PNH克隆在病程中继续扩增，少数患者的PNH克隆可能随时间的推移而变小。极少数患者PNH克隆甚至消失。因此，建议对所有PNH患者的克隆进行定期监测，若病情稳定，可每年监测1次；出现任何临床或血液学参数变化时应缩短监测间隔。亚临床的PNH应每6～12个月进行PNH克隆监测 [17],[20]。

二、C5补体抑制剂治疗过程中的监测

所有的补体抑制剂都需要长期甚至终身使用，其监测过程漫长而复杂。依库珠单抗是第一代末端补体C5抑制剂，2007年上市，是目前应用最为广泛、随访时间最长的补体抑制剂，欧美国家的临床应用经验丰富。C5补体抑制剂使用后，除监测临床表现的变化外，还需监测实验室指标、PNH克隆变化、补体活性等[21]。

（一）LDH、网织红细胞、血清铁蛋白监测

血清LDH水平降低通常与IVH减少相关，但与PNH无关的其他因素如感染、细胞坏死、心力衰竭等也会影响LDH水平的变化。在开始依库珠单抗治疗时，需监测LDH，LDH下降提示补体的末端活性被抑制。补体抑制剂使用后LDH明显下降，但后期发现LDH再次明显上升，且能除外其他因素，需考虑BTH的可能[3],[21]。少数情况下，补体抑制剂治疗前LDH并没有升高（如没有溶血时的血栓形成），可通过50％溶血补体检测（CH50）评估疗效。

网织红细胞是成熟红细胞的前体，在溶血加重时也会代偿性增加。应监测补体抑制剂治疗期间PNH患者绝对网织红细胞计数的变化，这有助于评估治疗反应和监测溶血事件的恢复情况[5]–[6]。

另外，在C5补体抑制剂使用前，患者由于长期的IVH，常合并铁缺乏或缺铁性贫血，在长期使用依库珠单抗后，EVH得到控制，可能出现铁过载，需要监测血清铁蛋白，必要时启动去铁治疗[21]–[22]。

（二）PNH克隆监测

C5补体抑制剂使用后，通过流式细胞术定期监测PNH克隆大小非常重要，建议在前2年至少每6个月检测PNH克隆；此后疾病稳定且接受稳定治疗时，可延长到每年1次[17]。

一般情况下，使用C5 补体抑制剂后，由于受到保护，PNH患者的红细胞PNH克隆增加。粒细胞和单核细胞的PNH克隆在治疗前往往较高，治疗后也会缓慢增加[14]。但在长期随访中，有些患者长期使用依库珠单抗后出现克隆自发减少，尽管如此，很少患者能够停止治疗，除非持续监测显示患者克隆明显减少，且没有明显的溶血证据（目标通常是至少2次连续的流式评估，确认PNH粒细胞和单核细胞的比例<10％）[21]。在此种情况下，停药后仍需动态监测PNH克隆，若出现PNH克隆细胞比例增加，可能需要重新开始治疗。在极少数情况下，PNH克隆变化可能预示着病态造血或白血病转化，此时需要通过全面的骨髓检查来进行评估。

（三）补体活性检测

血浆中的游离C5也可用作末端补体活性的生物标志物，其水平预计会因末端补体抑制而降低。游离C5水平可以帮助确定个体BTH事件是否与不完全的补体抑制相关。长期使用C5抑制剂，还可能出现EVH，EVH仅在使用C5抑制剂后出现。通过流式细胞仪测量细胞表面C3沉积，可帮助判断。PNH红细胞膜上的C3片段沉积，是C5抑制后反映上游补体活性的重要标志物，可作为EVH的检测指标。同时，使用Coombs试验评估红细胞上的C3沉积也可以作为一种替代方法[23]–[26]。

（四）依库珠单抗疗效不佳时的诊断及处理

1. 短期疗效不佳：应用依库珠单抗治疗6周后，如果LDH依然无明显改变，需检测C5补体多态性。值得注意的是，只有出现C5多态性时，才是真正意义上对依库珠单抗的“无反应”或“反应不佳”[7]。而在实际工作中，许多有残存贫血者被定义为“无反应者”或“反应不佳者”，这是误解。另外，20％的患者在使用依库珠单抗时需要调整剂量。

6周的常规治疗后，如果LDH<1.5 ULN，说明IVH得到控制，可按原剂量继续治疗。如果LDH≥1.5 ULN，但CH50显示补体被完全阻断，也可以继续治疗。此时，需要除外其他原因导致的LDH升高，尤其是LDH从非红细胞的其他组织（如肝、骨骼和心肌等）释放的原因，可以检测LDH同工酶加以鉴别。如果LDH≥1.5 ULN，且CH50显示补体没有被阻断，则考虑补体抑制剂剂量不足，可以提高剂量或转换为其他补体抑制剂。近年来，新型补体抑制剂不断获批上市，尤其是近年来开展临床试验的近端补体抑制剂，有望解决依库珠单抗疗效不佳的问题[23]–[28]。

2. BTH：主要表现为IVH和经典PNH症状的再次出现[24]。BTH是补体抑制剂效果不佳或失去疗效的表现，对有良好治疗反应（LDH<1.5 ULN、补体活性被完全抑制）者，有时可观察到间断的突破性IVH（血红蛋白下降、LDH升高、PNH症状再次发作），应考虑是否存在一些因素（如感染、药物等）激活或放大了补体的活性。此时不建议终止补体抑制剂治疗，因为一旦停药，会出现一系列溶血相关的并发症。BTH的病因具体鉴别方式见表2。可以通过控制诱发因素、继续补体抑制剂但增加剂量来改善，并密切监测患者的情况[27]–[30]。此外，出现BTH，如当前使用C5 抑制剂，可以转化为近端补体抑制剂。或在提高C5 抑制剂剂量仍无效时，转为近端补体抑制剂[28]。

**表2:** C5补体抑制剂使用后突破性溶血（BTH）事件的鉴别

类型	发生时间	发生比例	伴随事件	游离C5	补体抑制剂药物浓度	机制	干预
药效BTH	距离前次给药7~10 d	较常见	无	升高	不足	C5转化酶激活	增加剂量或给药方式调整
药代动力学BTH	任何时间	散发	感染、手术、应激、妊娠等	升高	不足	补体大量活化，C5抑制相对不足	积极控制伴随事件，增加给药剂量
血管外溶血	任何时间	较少见	脾大、Coombs 试验C3d阳性	正常	正常	C3调理红细胞	轻中度溶血，观察或更换上游补体抑制剂

3. EVH：未经治疗的PNH主要以IVH为特征，所有PNH患者都有不同程度的IVH。EVH通常发生在接受C5抑制剂治疗的患者中。患者接受C5抑制剂治疗后，红细胞不再被补体攻击，C3片段沉积在细胞膜上，从而导致红细胞在脾脏被破坏，发生EVH。使用C5抑制剂后，IVH被控制，但仍然发生持续贫血、黄疸加重、网织红细胞计数明显升高、脾大等表现的患者，应警惕EVH，可以检测C3沉积、抗人球蛋白试验、免疫指标等。部分C5抑制剂治疗的患者会出现直接抗球蛋白试验（抗C3d）阳性，可使用糖皮质激素、免疫抑制剂等，脾切除术在部分患者有效。目前，已经获批或正在开展临床试验的近端补体抑制剂，不仅能避免EVH的出现，还能对C5抑制剂使用后出现的EVH实现良好的控制[21],[28]–[30]。

4. C5补体抑制剂后的贫血：在C5补体抑制剂使用期间，部分患者血红蛋白并不能恢复正常。贫血的原因，除了IVH控制不满意、EVH以外，还有多种因素，应进行鉴别[29]。如在使用补体抑制剂时，网织红细胞计数较低或正常，考虑合并骨髓衰竭，则应在使用补体抑制剂同时，对伴随的骨髓衰竭进行诊断和治疗。叶酸缺乏症，是导致持续贫血的另一个常见原因，可通过补充叶酸来纠正。对伴有肾功能不全、与贫血程度不匹配的促红细胞生成素（EPO）降低患者，以及“低危”MDS，且EPO<500 U/L患者，可能需要外源性EPO。门静脉血栓导致的门脉高压、脾功能亢进（尤其是布加综合征后），可导致贫血和血小板减少，消化道出血会进一步加重贫血，可对症处理。既往反复输血导致的同种异体抗体导致的溶血性贫血、潜在MDS导致的无效红细胞生成等，都可能是贫血的原因[5]–[6],[27],[31]。

中国PNH的治疗即将步入补体抑制剂时代，谨慎地甄别各种临床情况，并给予恰当的治疗至关重要。当然，未来尚需累积更多的诊治经验对患者进行更精准的管理。
